# Identification of pulmonary adenocarcinoma and benign lesions in isolated solid lung nodules based on a nomogram of intranodal and perinodal CT radiomic features

**DOI:** 10.3389/fonc.2022.924055

**Published:** 2022-09-06

**Authors:** Li Yi, Zhiwei Peng, Zhiyong Chen, Yahong Tao, Ze Lin, Anjing He, Mengni Jin, Yun Peng, Yufeng Zhong, Huifeng Yan, Minjing Zuo

**Affiliations:** Department of Radiology, The Second Affiliated Hospital of Nanchang University, Jiangxi, China

**Keywords:** radiomics, computed tomography, solitary pulmonary nodule, lung adenocarcinoma, nomogram

## Abstract

To develop and validate a predictive model based on clinical radiology and radiomics to enhance the ability to distinguish between benign and malignant solitary solid pulmonary nodules. In this study, we retrospectively collected computed tomography (CT) images and clinical data of 286 patients with isolated solid pulmonary nodules diagnosed by surgical pathology, including 155 peripheral adenocarcinomas and 131 benign nodules. They were randomly divided into a training set and verification set at a 7:3 ratio, and 851 radiomic features were extracted from thin-layer enhanced venous phase CT images by outlining intranodal and perinodal regions of interest. We conducted preprocessing measures of image resampling and eigenvalue normalization. The minimum redundancy maximum relevance (mRMR) and least absolute shrinkage and selection operator (lasso) methods were used to downscale and select features. At the same time, univariate and multifactorial analyses were performed to screen clinical radiology features. Finally, we constructed a nomogram based on clinical radiology, intranodular, and perinodular radiomics features. Model performance was assessed by calculating the area under the receiver operating characteristic curve (AUC), and the clinical decision curve (DCA) was used to evaluate the clinical practicability of the models. Univariate and multivariate analyses showed that the two clinical factors of sex and age were statistically significant. Lasso screened four intranodal and four perinodal radiomic features. The nomogram based on clinical radiology, intranodular, and perinodular radiomics features showed the best predictive performance (AUC=0.95, accuracy=0.89, sensitivity=0.83, specificity=0.96), which was superior to other independent models. A nomogram based on clinical radiology, intranodular, and perinodular radiomics features is helpful to improve the ability to predict benign and malignant solitary pulmonary nodules.

## Introduction

Lung cancer remains the most common cause of cancer-related mortality worldwide because of its hidden incidence and poor prognosis (1, 2). The widespread availability of low-dose spiral CT screening has helped to reduce lung cancer mortality (3), but an increasing number of lung nodules are being detected. The average prevalence rate of pulmonary nodules in the first low-dose CT screening is 30%, of which less than 5% are malignant nodules, and peripheral adenocarcinoma is the most common (4). Moreover, compared with ground glass and subsolid nodules, solitary solid pulmonary nodules are more benign, and the diagnosis of benign and malignant nodules is more difficult (5, 6). According to the guidelines for the management of pulmonary nodules (7, 8), stratified management and routine periodic follow-up review of detected pulmonary nodules are needed. For high-risk nodules with a difficult diagnosis of benign and malignant nodules, puncture biopsy is recommended. However, conventional CT stratification assessment is easily affected by human factors, with only a moderate degree of consistency (9, 10), which may lead to misclassification and treatment of some patients as well as more radiation doses and psychological trauma. Needle aspiration biopsy of small nodules is more difficult and prone to misdiagnosis and pneumothorax (11), and some older patients with underlying diseases are not suitable for such invasive operations. F-18 fluorodeoxyglucose (FDG) PET-CT scan is highly sensitive and can play a key role in the identification of benign and malignant lesions. However, it is limited by its resolution and the inert state of some small lung cancer nodules, as it has no differential effect on nodules below 10 mm (12–14).

As a noninvasive new technique, radiomics can extract features with high throughput for analysis. It has been widely used in many aspects, such as differentiation of benign and malignant pulmonary nodules (15–19), invasion and metastasis (20, 21), histological classification (22), gene expression (23), and treatment prognosis (24). The classification of benign and malignant pulmonary nodules, in particular, have achieved excellence in radiomics, from purely benign-malignant differentiation to differentiation with inflammatory granulomas (15), tuberculous granulomas (19), and cryptococcal infections (17). However, most of the studies have only focused on the interior of the nodules.

As the microenvironment for nodular growth, the perinodular area has different degrees of heterogeneity, which is thought to be related to biological behaviors, such as the growth, blood supply, and invasion of the lesion (25, 26). Beig et al. (27) also showed that different cell tissue components in the perinodular area have different radiological characteristics. The purpose of this study was to construct a better clinical prediction model based on the radiomics characteristics of intra- and perinodular areas, which could provide more help for the identification and management of solitary solid pulmonary nodules.

## Materials and methods

### Patients

As this study was retrospective, the requirement for informed patient consent was waived, and this study was approved by the hospital ethics committee. We retrospectively collected 365 patients with benign lung nodules from 2020.1 to 2021.12 and 465 patients with peripheral-type adenocarcinoma from 2020.7 to 2021.12. The inclusion criteria were peripheral-type adenocarcinoma and benign lung lesions confirmed by surgical pathology. The patients were also screened according to the following exclusion criteria: 1) lesions larger than 30 mm or more than 1; 2) pure ground glass and subsolid nodules (with ground glass components inside); 3) lesions containing calcifications or small surrounding satellite foci; 4) no or poor quality thin-section enhanced CT images of the chest within 2 weeks before surgery; and 5) previous or current history of a malignant tumor.

**Figure 1:**
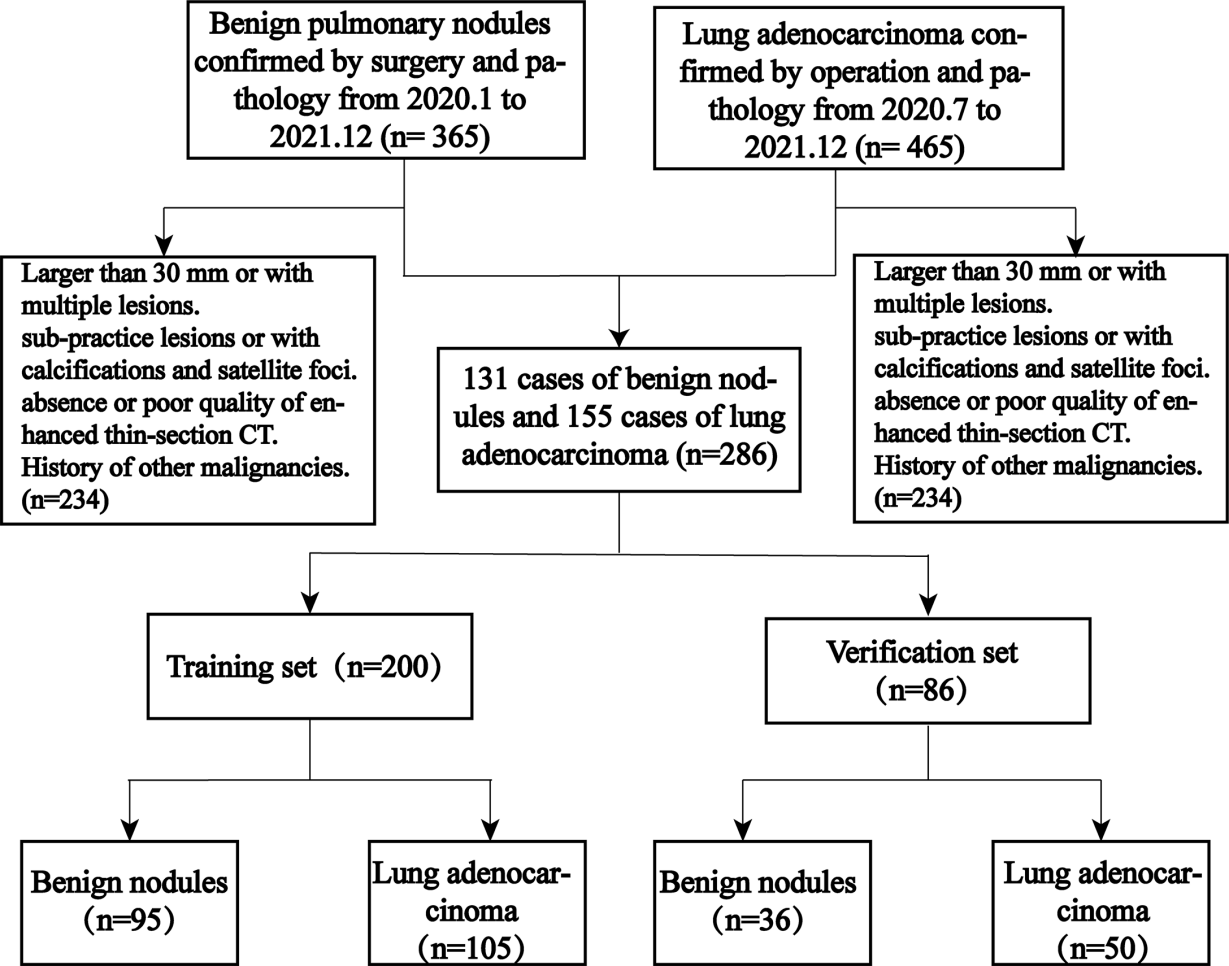
Patient stowage flowchart divided into training and validation sets at a ratio of 7:3.

As shown in **Figure 1**, 286 patients were eventually enrolled, including 155 cases of peripheral adenocarcinomas and 131 cases of benign lesions. Most of the benign lesions were granulomatous, including tuberculous granulomas (62, 47.3%), chronic inflammatory nodules (38, 29.0%), fungal granulomas (17, 12.9%), malignant tumors (9, 6.9%), and sclerosing alveolar cell tumors (5, 3.8%). All patients were divided into a training set and validation set at 7:3, including a training set (105 cases of adenocarcinoma, 95 cases of benign lesions) and a verification set (50 cases of adenocarcinoma and 36 cases of benign lesions).

### CT image acquisition and visual image review

All patients underwent chest enhanced CT 2 weeks before the operation, and all examinations were performed on 3 CT scanners: 1) GE Revolution HD CT; 2) SOMATOM Definition Flash; 3) Philips 256iCT. The scanning method was as follows: using a double-barrel high-pressure syringe, contrast medium (Ioversol, 350 mg I/ml) was injected into the right elbow vein at a rate of 3.5 ml/s. The contrast medium dosage was 1.2-1.5 mL/kg. The images of the artery, portal vein, and balance phase were obtained after 25 s, 55 s, and 90 s, respectively. The relevant scanning parameters are shown in **Supplementary Table 1**.

Two senior diagnostic thoracic radiologists evaluated the chest CT images separately under the lung window (window width = 1500 HU, window position = - 450) without knowledge of pathology and discussed a unified opinion when there was a difference of opinion. The evaluation included: 1) location (transverse, longitudinal); 2) size (long diameter, short diameter, mean diameter); 3) border (clear, indistinct); 4) lobulation; 5) burr; 6) cavity; 7) bronchial inflation; 8) vascular abnormality; 9) pleural traction; 10) pleural effusion; and 11) lymph node enlargement. The horizontal location was defined as whether the lesion was located under the pleura, and the vertical location was defined as the lung lobe where the lesion was located. The size was defined by the largest cross-section of the lesion, the lobulation was defined as an uneven and bumpy surface of the lesion, the burr was defined as a spine-like protrusion of 2 mm or more on the surface of the lesion, bronchial insufflation was defined as an air-containing bronchial shadow within or at the edge of the lesion, vascular abnormality was shown as an abnormal aggregation or dilatation, pleural pull was defined as a depression of the pleura adjacent to the lesion or lymph node, and enlargement was defined as a short diameter greater than 10 mm and without calcification (28). All visual CT image evaluation components and clinical information were collectively referred to as clinical radiology (C-R) for analysis.

### Nodule segmentation and feature extraction

All nodules were segmented manually by a researcher (A) using the open-source software 3D Slicer (version 4.8.1) (https://www.slicer.org/) on the lung window (window width = 1500 HU, window level =-450) until the entire lesion was sketched. The nodule region was first outlined to form the intranodal region of interest (intra-ROI) and then expanded outward by 5 mm using the 3D Slicer semiautomatic segmentation program (27) to form the perinodal region of interest (peri-ROI), and all unrelated large vessels, bronchi, and chest wall tissue were manually removed.

**Figure 2:**
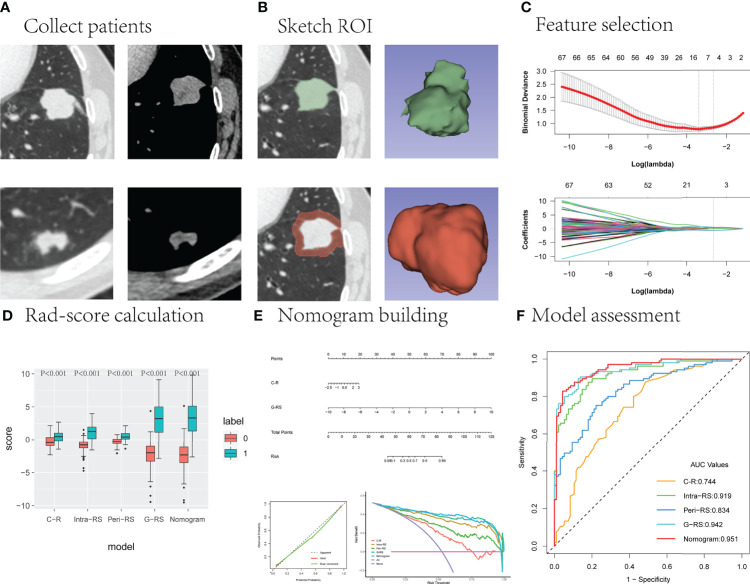
The main research process. **(A)** The upper and lower layers are CT images of the lung window and mediastinal window in patients with lung cancer and benign lung nodules, respectively. **(B)** Outline of the intranodal and perinodal areas in a patient with lung cancer. **(C)** Useful radiomics features are filtered from the high-dimensional features. **(D–F)** The performance of the different models is compared and the best model is selected to construct column line graphs and evaluate clinical effectiveness.

The PyRadiomics program on the 3D Slicer was used to automatically extract ROI radiomics features and resample the images. In total, 851 features were extracted from intranodular and perinodular areas, including 107 original features and 744 wavelet features. Original features included shape (14), first-order statistics (18), gray-level cooccurrence matrix (glcm, 24), gray-level dependence matrix (gldm, 14), gray level run length matrix (glrlm, 16), gray level size zone matrix (glszm, 16), and neighborhood gray-tone difference matrix (ngtdm, 5). All features have been uploaded to the supplementary material, and the main study flow is shown in **Figure 2**.

### Reproducibility assessment

After 30 days, images of 30 patients in the cohort were randomly selected, and researchers A and B adopted the same method and process for nodule segmentation and feature extraction, respectively. They were completely unaware of each other’s segmentation process. Intra- and intergroup correlation coefficients (ICCs) were calculated to assess the repeatability and stability of nodule segmentation and feature extraction. An ICC > 0.75 indicated good reproducibility.

### Feature selection and radscore calculation

All patients were randomly divided into a training cohort and a validation cohort at a ratio of 7:3, and group randomization was assessed using 200 replicate tri-fold cross-validation. Before feature selection, intra- and perinodal features with all nodules were normalized using Z score [(x - μ)/σ]. Feature selection was performed using the following steps: 1) intra- and interobserver agreement was assessed for all features, and those with ICC values greater than 0.75 were selected for the next step; 2) the correlation coefficient was calculated to remove redundant features (Pearson for normal distribution, not Spearman); 3) the minimum redundancy maximum relevance (mRMR) method was used to rank features according to their relevance-redundancy index to rank the features, and the top 100 features were selected; 4) finally, the 10-fold cross-validation Lasso method was used to filter out the final features. The radscores of intranodular (intra-RS) and perinodular (peri-RS) lesions were calculated according to their coefficients.

The screened intranodal and perinodal features were combined to build a gross radiomic model (G-RS) using forward stepwise selection multiple logistic regression, and the gross radiomic labels were calculated based on the weighting coefficients.

### Screening of clinical radiological features and construction of the nomogram

Univariate and multivariate analyses were used to screen the clinical-radiological features and build C-R models. Clinical label scores were calculated based on the weighting coefficients. The screened clinical radiological features were combined with the gross radiomic labels, and column line graphs were constructed by multiple logistic regression.

### Model performance evaluation and verification

The area under the receiver operating curve (AUC value) and its associated metrics (sensitivity, specificity, accuracy, positive predictive value, negative predictive value) were used for model performance assessment, the Hosmer–Lemeshow test was used to assess the degree of model fit, and the clinical decision curve (DCA) was used for model clinical utility assessment.

### Statistical analysis

All statistical analyses were performed using R 4.11 (http://www.r-project.org) and SPSS 25.0 (IBM, Armonk, NY, USA) software. The “glmnet” package was used for lasso logistic regression to filter features and multiple logistic regression to build models; the “rms” package was used for drawing nomograms and calibration curves, and the “pROC” package was used for plotting ROC curves and calculating AUC values and related indicators. The Delong test was used for comparison between models, and the Akaike information criterion (AIC) was used for model ranking and selection. Two-sided p values < 0.05 indicate statistical significance.

## Results

### Clinical radiological features

A total of 155 cases of peripheral adenocarcinoma (76 males and 79 females, mean age 62.3 ± 8.9 years [range 40-85 years]) and 131 cases of benign nodules (92 males and 39 females, mean age 54.6 ± 11.9 years [range 22-86 years]) were included in this study. In the training set, there were significant differences between the lung cancer and benign nodule groups in terms of sex, age, short diameter, mean diameter, border, lobulation, burr, vascular abnormality, pleural traction, and lymph node enlargement. In the validation set, there were significant differences in age, long diameter, short diameter, mean diameter, lobulation, burr, bronchial inflation, vascular abnormality, and pleural traction (P<0.05) **(** **Table 1** **)**.

**Table 1:** Clinical-radiological performance of patients in the training and validation sets.

Characteristics	Training set			validation set		
	Lung Cancer (n = 105)	Benign nodules (n = 95)	p-value	Lung Cancer (n = 50)	Benign nodules (n = 36)	p-value
**Age**	62.8 ± 9.3	54.8 ± 12.2	<0.001	61.0 ± 7.9	54.1 ± 11.1	0.001
**Sex**			0.001			0.117
Male	54 (51.4)	70 (73.7)		22 (44.0)	22 (61.1)	
Female	51 (48.6)	25 (26.3)		28 (56.0)	14 (38.9)	
**Vertical position**			0.716			0.190
Upper right lung	37 (35.2)	27 (28.4)		16 (32.0)	9 (25.0)	
Right middle lung	9 (8.6)	7 (7.4)		3 (6.0)	5 (13.9)	
Lower right lung	21 (20.0)	26 (27.4)		7 (19.8)	10 (27.8)	
Upper left lung	19 (18.1)	19 (20.0)		9 (18.0)	7 (19.4)	
Lower left lung	19 (18.1)	16 (16.8)		15 (30.0)	5 (13.9)	
**lateral position**			0.527			0.662
Subpleural	55 (52.4)	54 (56.8)		26 (52.0)	17 (47.2)	
Non-Pleural	50 (47.6)	41 (43.2)		24 (48.0)	19 (52.8)	
**Long Diameter**	17.9	16.4	0.074	19.4	15.5	0.003
**Short Diameter**	15.4	13.3	0.004	16.1	13.2	0.006
**Average diameter**	16.7	14.9	0.020	17.8	14.4	0.003
**Boundary**			0.045			0.421
Clear	73 (69.5)	53 (55.8)		35 (70.0)	28 (77.8)	
Blur	32 (30.5)	42 (44.2)		15 (30.0)	8 (22.2)	
**Lobulation**			0.003			0.075
Present	100 (95.2)	78 (82.1)		49 (98.0)	32 (88.9)	
Absent	5 (4.8)	17 (17.9)		1 (2.0)	4 (11.1)	
**Speculation**			0.006			<0.001
Present	97 (92.4)	75 (78.9)		48 (96.0)	23 (63.9)	
Absent	8 (7.6)	20 (21.1)		2 (4.0)	13 (36.1)	
**Cavity**			0.376			0.584
Present	19 (18.1)	22 (23.2)		6 (12.0)	3 (8.3)	
Absent	86 (81.9)	73 (76.8)		44 (88.0)	33 (91.7)	
**Air bronchogram**			0.142			0.046
Present	13 (12.4)	19 (20.0)		2 (4.0)	6 (16.7)	
Absent	92 (87.6)	76 (80.0)		48 (96.0)	30 (83.3)	
**Vascular abnormal**			0.005			0.005
Present	53 (50.5)	29 (30.9)		29 (58.0)	10 (27.8)	
Absent	52 (49.5)	65 (69.1)		21 (42.0)	26 (72.2)	
**Pleural pull**			<0.001			0.009
Present	80 (76.2)	49 (51.6)		35 (70.0)	15 (41.7)	
Absent	25 (23.8)	46 (48.4)		15 (30.0)	21 (58.3)	
**Pleural effusion**			0.212			0.813
Present	4 (3.8)	1 (1.1)		1 (2.0)	1 (2.8)	
Absent	101 (96.2)	94 (98.9)		49 (98.0)	35 (97.2)	
**Lymph node enlarge**			0.016			0.899
Present	20 (19.0)	7 (7.4)		6 (12.0)	4 (11.1)	
Absent	85 (81.0)	88 (92.6)		44 (88.0)	32 (88.9)	

### Construction of the clinical radiology model

The univariate analysis showed significant differences (P<0.05) in age, sex, mean diameter, border, burr, vascular abnormality, pleural pull, and lymph node enlargement (**Table 2**), which were substituted into the multivariate analysis, showing significant differences in age and sex. The C-R model was constructed, and the signature score was calculated. The AUC values of the training set and verification set were 0.744 and 0.698, respectively, and the accuracy was 0.70 and 0.65.

**Table 2:** Univariate and multifactorial analysis of clinical radiological characteristics.

	Univariate			Multifactorial		
	OR	CI	P	OR	CI	P
Age	0.93	0.91-0.96	<0.001	0.94	0.91-0.97	<0.001
Sex	0.38	0.21-0.69	<0.001	0.29	0.14-0.59	<0.001
Average diameter	0.94	0.89-0.99	0.02	NA	NA	NA
Boundary1	1.81	1.01-3.23	0.05	NA	NA	NA
Lobulation1	0.31	0.13-0.74	0.01	NA	NA	NA
Vascular abnormal	0.44	0.24-0.78	0.01	NA	NA	NA
Pleural pull	0.33	0.18-0.61	<0.001	NA	NA	NA
Lymph node enlarge	0.34	0.14-0.84	0.02	NA	NA	NA

OR, Odds Ratio; CI, confidence interval.

### Repeatable quantization

Intra- and interobserver consistency analysis of intra- and perinodular features showed that there were 802 features with ICC ≥ 0.75 (94.2%) in nodules and 788 features with ICC ≥ 0.75 (92.6%) around nodules (**Supplementary Figure 1**).

### Construction and verification of the radiomics signature

**Figure 3:**
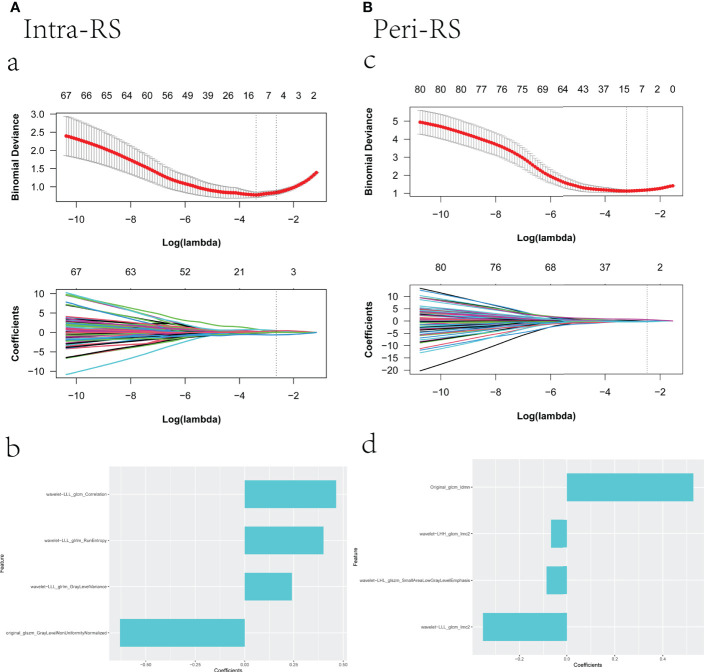
Screening radiomics features using lasso; (a): vertical dashed line indicates the best model fitted when λ = 0.070 and Log(λ) equals -2.648, (c): vertical dashed line indicates the best model fitted when λ = 0.084 and Log(λ) equals -2.471; (b), (d) are the selected feature weight coefficients.

After removing poorly reproducible and redundant features, the features were sorted using mRMR, and the top 100 features were ultimately selected for lasso screening. Four features were retained for both intra- and peri- nodular (17), and Intra-RS and Peri-RS models were established (**Figure 3**). The G-RS model was built for the combined intra- and nodular features using forward stepwise multiple logical regression.

**Figure 4:**
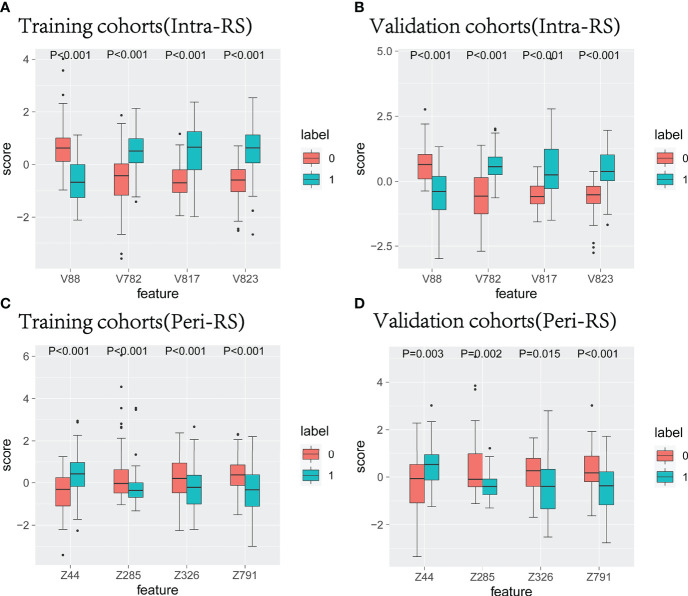
The characteristic box line map in the training set and verification nodule **(A, B)** and around the nodule **(C, D)**. 0 represents benign nodules, 1 represents lung cancer, V represents radiological features in nodules, and Z represents radiological features around nodules.

All intranodal and perinodal features ultimately preserved were significantly different between the lung cancer group and the benign nodule group (P<0.05), which could identify lung cancer and benign nodules (**Figure 4**).

**Figure 5:**
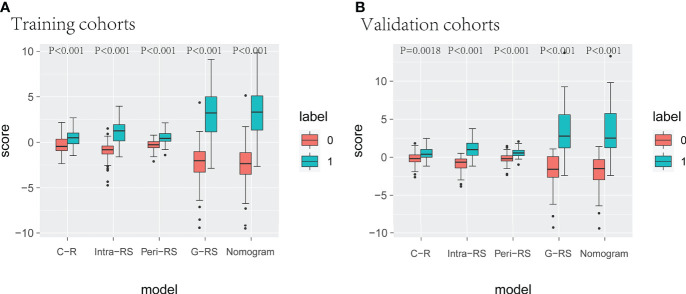
Box line plots for different model radscores. **(A)** (training set), **(B)** (verification set), 0 for benign nodules, 1 for lung cancer.

Radscore of C-R, Intra-RS, and Peri-RS models were calculated based on feature weight coefficients, with the relevant feature names and formulas for calculating radiomics scores shown in **Supplementary Table 2**. Box plots showed a significant difference in all model scores between the lung cancer and benign nodule groups in both the training and validation sets (P<0.05) (**Figure 5**).

**Figure 6:**
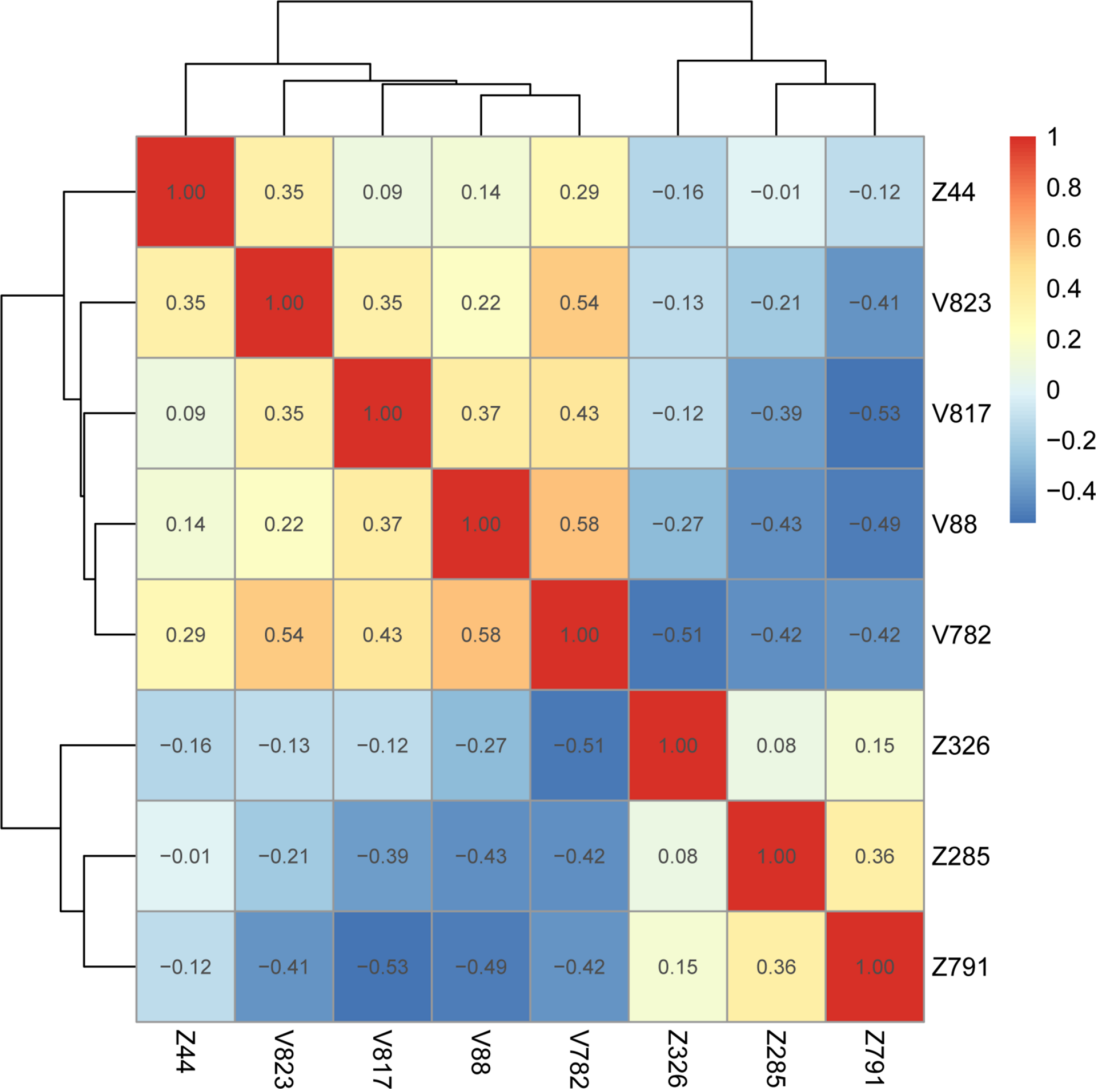
Gross radiology model feature correlation heatmap. V represents intranodular features, and Z represents peri-nodular features (**Supplementary Table 2**).

Heatmaps of correlation coefficients for all retained features showed that the correlation coefficients between intranodal and perinodal and total radiomic features were less than 0.75, which suggested no covariance between these features (**Figure 6**, **Supplementary Figure 2**).

The AUC values of the G-RS model (training set: 0.942; validation set: 0.934) were higher than those of either the intra-RS (training set: 0.919; validation set: 0.911) or Peri-RS (training set: 0.835; validation set: 0.805) model alone. The AUCs and related metrics of the other models are shown in **Table 3**. The mean AUC values of the five models after cross-validation of all patients were: 0.722 (C-R), 0.912 (Intra-RS), 0.835 (Peri-RS), 0.926 (G-RS), and 0.947 (Nomogram), which were very close to the previous results, indicating the randomized nature of the grouping in this study. The Intra-RS, Peri-RS, and G-RS ROC curves are shown in **Supplementary Figure 3**.

**Table 3:** Prediction performance of the five models in the training and validation sets.

	AUC	95%CI	Sensitivity	Specificity	Accuracy	PPV	NPV
**Training set**							
C-R	0.744	0.67-0.81	0.87	0.51	0.70	0.67	0.79
Intra-RS	0.919	0.88-0.95	0.87	0.82	0.85	0.84	0.85
Peri-RS	0.835	0.78-0.88	0.75	0.78	0.76	0.79	0.74
G-RS	0.942	0.91-0.97	0.89	0.87	0.88	0.88	0.98
Nomogram	0.951	0.92-0.97	0.83	0.96	0.89	0.96	0.83
**Validation set**							
C-R	0.698	0.58-0.81	0.60	0.72	0.65	0.75	0.57
Intra-RS	0.911	0.85-0.97	0.70	0.97	0.81	0.97	0.70
Peri-RS	0.805	0.70-0.90	0.88	0.67	0.79	0.79	0.80
G-RS	0.934	0.88-0.98	0.78	1.00	0.87	1.00	0.77
Nomogram	0.941	0.90-0.98	0.90	0.86	0.88	0.90	0.86

AUC, area under the receiver operator characteristic curve; 95%CI, 95% confidence interval; PPV, positive predictive value; NPV, negative predictive value.

### Construction and calibration of nomogram

**Figure 7:**
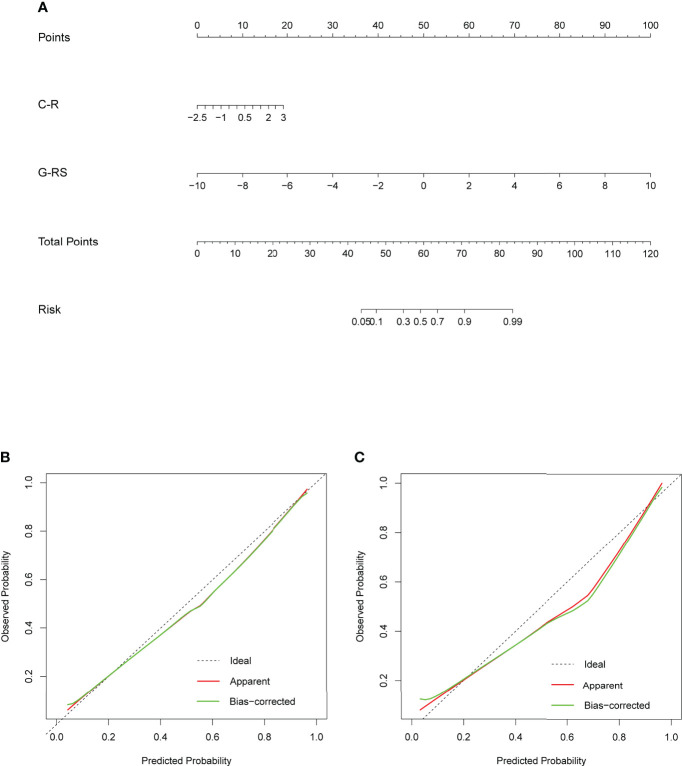
**(A)** Nomogram based on clinical radiology and gross radiomics. Calibration curves of the nomogram in the training **(B)** and validation **(C)** sets; the x-axis indicates the predicted probability estimated by the nomogram, while the y-axis indicates the actual probability. Apparent probabilities and bias-corrected probabilities are indicated by red and green solid lines, respectively.

To develop a high predictive performance model with clinical applicability, a multivariate logistic regression-based analysis showed C-R (P=0.015, 95% CI: 1.14-3.28) and G-RS (P<0.001, 95% CI: 1.84-3.54) as independent influencing factors for prediction (**Supplementary Table 3**), which were combined into a nomogram (**Figure 7**).

**Figure 8:**
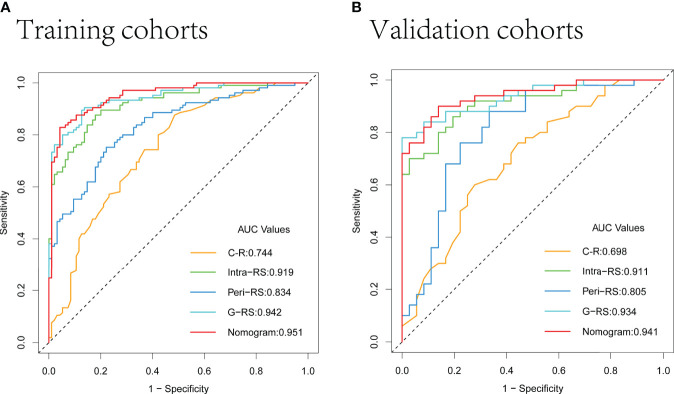
ROC curves of the 5 models in the training **(A)** and validation **(B)** sets.

The AUC values of the nomogram, which consisted of C-R and G-RS in the training and validation sets, were as follows: training set = 0.95, validation set = 0.94 (**Figure 8**). These were higher than those of the C-R model (training set = 0.74, validation set = 0.68), Intra-RS model (training set = 0.91, validation set = 0.91), Peri-RS model (training set = 0.83, validation set = 0.80) and G-RS model (training set = 0.94, validation set = 0.93). The accuracy, sensitivity, specificity, positive predictive value, and negative predictive value of the nomogram were higher than 80% (**Table 3**).

The Delong test showed significant differences between the nomogram and the C-R, intra-RS, and Peri-RS models (P< 0.05). There was no significant difference between the G-RS model and the nomogram, but the nomogram had the smallest AIC value (116.79) of all models (**Supplementary Table 4**). The calibration curves of the nomogram are shown in **Figure 7**, and the Hosmer–Lemeshow test showed no statistical significance in the training set or validation set (p values of 0.657 and 0.938), indicating that the nomogram had a good fit.

In addition, to understand the efficacy of the nomogram in different sex, age, and scan model case groups, we conducted separate subgroup studies, which showed no significant difference in its predictive efficacy between the abovementioned different groups and the total cohort (**Supplementary Figure 4**), and the Delong test p values were all >0.05 (**Supplementary Table 5**).

**Figure 9:**
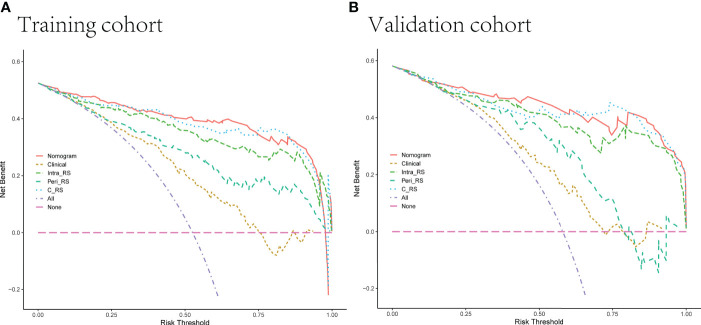
**(A, B)** Decision curves of 5 models in the training and validation sets. The net income is shown on the y-axis, and the probability threshold is shown on the x-axis.

The decision curve DCA (**Figure 9**) showed more net clinical benefit for the Intra-RS, Peri-RS, G-RS, and nomogram than the C-R model for most threshold ranges, and the nomogram obtained the most net clinical benefit.

## Discussion

In this study, we analyzed the intranodal and perinodal regional radiomic features and found that the combined gross radiomic model was better than the single intranodular or perinodular model, which proved the superposition of perinodular features. Finally, a nomogram model based on clinical radiology and gross radiomics was constructed and tested, which improved the ability to distinguish between benign and malignant solitary pulmonary nodules. The DCA decision curve demonstrated its good clinical utility.

The differentiation of benign and malignant pulmonary nodules has always been a difficult problem for radiologists. With the popularization of screening and improvement of treatment, the assessment of lesion size, density, location, and shape by conventional CT can no longer meet the requirements of patients and clinicians. Just like in the multifactorial analysis of the clinical-radiological features in the current study, only two high-risk factors, gender and age, were screened out. Consistent with previous studies (29), it was shown that isolated solid nodules of the lung are more difficult to diagnose correctly by conventional CT evaluation, as most benign nodules are associated with malignant CT signs such as lobarization, burr, and pleural traction. Patients with lung adenocarcinoma have a higher proportion of women and the probability of prevalence increases accordingly with age. Radiomics can extract focus information with high throughput and can be combined with clinical and conventional imaging manifestations and laboratory indicators, which greatly improves the diagnostic efficiency. However, most studies have not focused on the peritumor region (15–18). Chen et al. (15) constructed an radiomics model to identify adenocarcinoma and granulomatous nodules in the lung with good performance, while Liu et al. (16) included different categories of benign nodules and showed that the diagnostic performance of the radiomics model was better than that of the Lung-RADS model, and the diagnostic performance of LDCT-based radiomic models to differentiate adenocarcinomas from benign lesions in solid pulmonary nodules were equivalent to that of standard-dose CT (17). constructed a clinical-radiomics model to identify pulmonary cryptococcosis and pulmonary adenocarcinoma, screening four visual radiological features of maximum diameter, size, lobulation and pleural retraction, consistent with our routine CT evaluation, including 24 radiomics features of different categories, with wavelet features as the main part (19/24), quantifying the heterogeneity of lesions of different grades that are not recognized by human eyes. The model constructed by Marmor et al. (30) incorporated clinical and laboratory indices, but the AUC was only 0.76.

In the past decade, the central role of the tumor microenvironment(TME) in the development and progression of primary lung cancer has been recognized (31, 32). Cancer cells are closely associated with the extracellular matrix (ECM), mesenchymal cells (e.g., fibroblasts), infiltrating immune cells, and the vascular system, an environment that in some cases is critical for tumorigenesis or growth, and in other cases can prevent tumorigenesis or even promote tumor growth. and recent studies on the radiomics aspects of the peritumoral region have intensified and yielded many results. Huang et al. (33) established a nomogram model to predict the pathological aggressiveness of isolated lung nodules based on clinical, intranodal, and perinodal radiomics. Vaidya et al. (34) developed an intra- and perinodal radiomic risk score and associated nomogram to predict disease-free survival (DFS) and adjuvant chemotherapy after surgery (ACT) efficacy in early-stage non-small-cell lung cancer. Zhu et al. (35) established a radiomic model to predict Ki-67 in early lung adenocarcinoma, and the results showed that a radiomic model combining intranodal and perinodal features was better than intranodal features alone. perrone et al. (36) studied the relationship among TME, radiomic features and pathological histological aspects in patients with the same stage and visual imaging presentation but significantly different survival cycles, showing that the inflammatory response in TME is a key determinant of cancer cell growth and proliferation, with a more pronounced role for NLRP3 inflammatory vesicles. And the differences in TME were closely related to the imaging histological features (two were textural features and four were intensity features). In terms of nodal benign-malignant discrimination, Beig, N et al. (27) showed that different pathological compositions of lung cancer nodules and benign perinodular regions exhibited different radiomic features,. The interface around the lung cancer has dense infiltrating lymphocytes and associated macrophages, which appear as smooth texture on CT images. In contrast, normal lung tissue and macrophages around the granuloma exhibit high expression of mid-frequency Gabor features, respectively. The high nucleoplasmic ratio within the tumor exhibited low-frequency Gabor features, Lin et al. (37) showed that deep learning models based on intranodal and perinodular radiomics outperformed single intranodal or perinodular models in lung cancer and granuloma discrimination.

In this study, the perinodular area was defined as nodular outward expansion of 5 mm. This was based on the study of Beig, N et al. (27), which demonstrated that the perinodal 5-mm region features had optimal discrimination performance. The study of Calheiros, J (38) showed that the perinodal region features had good stability but poor discrimination performance compared to the intranodal features, which may be because the intranodal area is the main tumor area and carries more substantive information. The results of this study are consistent with them, but the Peri-RS model still achieved an AUC value of 0.83 and an accuracy of 76%, which proves that it is of great help in the differential diagnosis as an integral part of the focus. The study of clinical-radiological performance through univariate and multivariate analysis ultimately included only two clinical variables, age and sex, and the AUC of the model was only 0.744, indicating its poor stability and predictive performance. The final nomogram improved the AUC value to 0.95 with an accuracy of 0.89, which was higher than all other models, although the Delong test showed no significant difference between the G-RS model and the nomogram, which may have been due to the low performance of the combined C-R model and no obvious effect on the final alignment map. We believe that the nomogram performs best when considering the relevant indices of AUC and AIC value.

The intranodular and perinodular features extracted in this study could well distinguish between the lung cancer group and the benign nodule group (P<0.05), which consisted of Glcm, Glrlm, and Glszm. Glcm is a texture feature that studies the spatial correlation of grayscale and represents the heterogeneity between images by calculating the correlation between two pixels in a certain distance and direction. Glrlm has information about the spatial distribution of consecutive pixels at the same gray level in one or more directions. As an advanced texture statistical feature, Glszm provides the size feature of the area around the grayscale and has good performance in characterizing texture consistency. The study by Chen et al. (15) also showed that the imaging histological features associated with GLRLM, GLCM are good for the identification of lung nodules, and this non-uniform intensity distribution of the run length (non-uniform intensity distribution), randomness in neighborhood intensity values (randomness (non-uniform intensity distribution of the run length), randomness in neighborhood intensity values) reflects the higher heterogeneity and more complex internal structure of lung adenocarcinoma. a study by Liu et al. (16) showed higher NGTDM_Strength values (slower image intensity changes) in benign lung nodules, indicating a more homogeneous internal structure. Other related studies have also proven the excellent performance of the above features in characterizing tumor heterogeneity (33, 39, 40).

In this study, intra- and interobserver agreement studies were performed for all radiomic features, and the proportion of ICC values greater than 0.75 exceeded 90% for all groups, which once again demonstrates the reproducibility and stability of radiomics in lesion segmentation and feature extraction (41). Both calibration curves and Hosmer–Lemeshow passed validation of the nomogram. Subgroup analysis proved that the final nomogram was not affected by age, sex, or different scanning models and showed its good generalization performance to some extent.

There are still some limitations in this study. First, because this was a retrospective study, there may have been biases in different directions, and this study avoided these possible biases by adopting a strict experimental procedure. Second, CT images from 3 different scanners may have some bad effects, which could have reduced but not eliminated biases by image resampling and data standardization preprocessing. Third, the benign group was included in this study. The variety of cases, which may have contained certain lesions with clear benign manifestations, had some influence on the construction of the model, and we tried to reduce these effects by collecting cases with strict exclusion criteria (calcifications, satellite foci, etc.) Finally, the data in this study were insufficient, and there was a lack of appropriate external verification queues; these are future directions of the study.

## Conclusion

In conclusion, this study proved the additional value of the perinodular area in the differential diagnosis of benign and malignant nodules and the reproducibility of radiological features. Finally, we constructed a nomogram based on clinical radiology, intranodal, and perinodal radiomic features, which achieved the highest predictive performance and verified its good stability. It can be used as a good noninvasive tool to help radiologists and clinicians distinguish between benign and malignant pulmonary nodules.

## Data availability statement

The original contributions presented in the study are included in the article/**Supplementary Material**. Further inquiries can be directed to the corresponding author.

## Ethics statement

The studies involving human participants were reviewed and approved by the Ethics Review Board of The Second Affiliated Hospital of Nanchang University. Written informed consent for participation was not required for this study in accordance with the national legislation and the institutional requirements.

## Author contributions

MZ and LY designed the study. LY and ZP collected and classified the data and established the models. ZC, YT and AH did the statistical analysis. ZL, MJ, YP, YZ and HY made substantial revisions to the manuscript. All authors contributed to the article and approved the submitted version.

## Funding

This study was supported by Jiangxi Province Applied Research Incubation Program (grant numbers 20212BAG70048) and Jiangxi Provincial Education Department Key Projects (grant number GJJ200106).

## Conflict of interest

The authors declare that the research was conducted in the absence of any commercial or financial relationships that could be construed as a potential conflict of interest.

## Publisher's note

All claims expressed in this article are solely those of the authors and do not necessarily represent those of their affiliated organizations, or those of the publisher, the editors and the reviewers. Any product that may be evaluated in this article, or claim that may be made by its manufacturer, is not guaranteed or endorsed by the publisher.
