# Supplementary material for: Identification of pulmonary adenocarcinoma and benign lesions in isolated solid lung nodules based on a nomogram of intranodal and perinodal CT radiomic features
Source: Front Oncol. 2022 Sep 6;12:924055. doi: 10.3389/fonc.2022.924055 (PMC9485677; doi:10.3389/fonc.2022.924055)
Supplement: Supplementary file 1 [file DataSheet_1.docx]

Supplementary Material

1. **Supplementary Table**

**Supplementary Table 1.** Relevant parameters of the CT equipment used

|  | **GE Revolution HD CT** | **SOMATOM Definition Flash** | **Philips 256 iCT** |
| --- | --- | --- | --- |
| CT tube voltage | 120 kVp | 120 kVp | 120 kVp |
| CT tube current | 180mAs | 150mAs | 150mAs |
| CT rotation time | 0.6s | 0.5s | 0.5s |
| Pitch | 0.984 | 1 | 0.758 |
| Layer thickness | 5mm | 5mm | 5mm |
| Reconstruction thickness | 1.25mm | 1mm | 1mm |
| Reconstruction pitch | 1.25mm | 1mm | 1mm |
| Reconstruction algorithm | ASIR-V, Level 4 | SAFIRE, Level 3 | i Dose 4,  Level 3 |
| Contrast agent | Ioversol | Ioversol | Ioversol |
| Contrast agent concentration | 350 mgI/mL | 350 mgI/mL | 350 mgI/mL |
| Contrast agent dosage | 1.2-1.5mL/kg | 1.2-1.5mL/kg | 1.2-1.5mL/kg |
| Contrast agent infused rate | 3.5ml/s | 3.5ml/s | 3.5ml/s |
| Venous phase interval time | 55s | 55s | 55s |
| Image matrix | 512*512 | 512*512 | 512*512 |

**Supplementary Table 2.** All model feature weighting coefficients and Radscore calculation formula

| **Model** | **Intercept/Feature Name** | **Regression coefficient** |
| --- | --- | --- |
| **Intra-RS** | **Intercept=0.1584** | **β** |
| V88 | original_glszm_GrayLevelNonUniformityNormalized | -0.6302 |
| V782 | wavelet-LLL_glcm_Correlation | 0.4638 |
| V817 | wavelet-LLL_glrlm_GrayLevelVariance | 0.2410 |
| V823 | wavelet-LLL_glrlm_RunEntropy | 0.4002 |
| **Peri-** **RS** | **Intercept=0.1109** | **β** |
| Z44 | Original_glcm_Idmn | 0.5273 |
| Z285 | wavelet-LHL_glszm_SmallAreaLowGrayLevelEmphasis | -0.0868 |
| Z326 | wavelet-LHH_glcm_Imc2 | -0.0680 |
| Z791 | wavelet-LLL_glcm_Imc2 | -0.3522 |
| **G-** **RS** | **Intercept=0.5978** | **β** |
| V88 | original_glszm_GrayLevelNonUniformityNormalized | -0.6477 |
| V782 | wavelet-LLL_glcm_Correlation | 1.0158 |
| V817 | wavelet-LLL_glrlm_GrayLevelVariance | 1.8870 |
| V823 | wavelet-LLL_glrlm_RunEntropy | -0.1954 |
| Z44 | Original_glcm_Idmn | 0.8188 |
| Z285 | wavelet-LHL_glszm_SmallAreaLowGrayLevelEmphasis | -0.3982 |
| Z326 | wavelet-LHH_glcm_Imc2 | -0.4589 |
| Z791 | wavelet-LLL_glcm_Imc2 | -0.5192 |
| **C-R** | **Intercept=-5.6930** | **β** |
| Age |  | 0.07271 |
| Sex | female | 1.1003 |
| **Intra-RS=**  **0.1584-** **V88×0.6302+V782×0.4638+V817×0.2410+V823×0.4002** | | |
| **Peri- RS=**  **0.1109+Z44×0.5273-Z285×0.0868-Z326×0.0680-Z791×0.3522** | | |
| **G- RS=**  **0.5978- V88×0.6477+V782×1.0158+V817×1.8870-V823×0.1954+Z44×0.8188-Z285×0.3982-Z326×0.4589-Z791×0.5192** | | |
| **C-R=**  **-5.6930+ Age×0.07271+ Sex×1.1003** | | |
| Intra-RS: intranodal radiomic score; Peri- RS: perinodal radiomic score; G- RS: intranodal plus perinodal radiomic score; C-R: clinical radiology score; V: intranodal features; Z: perinodal features; β: regression coefficient | | |

**Supplementary Table 3**. Multivariate logistic regression for clinical radiology and total radiomics models

|  | **OR** | **CI** | **P** |
| --- | --- | --- | --- |
| **C-R** | 1.93 | 1.14-3.28 | 0.015 |
| **Nomogram** | 2.56 | 1.84-3.54 | <0.001 |
| **OR:** Odds ratio; **CI**: Confidence interval. | | | |

**Supplementary Table 4.** Comparison of Delong test and AIC values of different models

|  | **Delong test p-value (compared with nomogram)** | **AIC** |
| --- | --- | --- |
| **C-R** | <0.001 | 243.18 |
| **Intra-RS** | 0.009 | 145.79 |
| **Peri-RS** | <0.001 | 203.07 |
| **G-RS** | 0.136 | 123.92 |
| **Nomogram** | NA | 116.79 |
| **AIC**: Akaike information criterion. | | |

**Supplementary Table 5.** Delong test for subgroup analysis

|  | **Gender (compared to total cohort)** | | **Age (compared to total cohort)** | | **Equipment (compared to total cohort)** | | |
| --- | --- | --- | --- | --- | --- | --- | --- |
|  | male | Female | ≥60 years old | <60 years old | GE | Siemens | Philips |
| **P** | 0.661 | 0.291 | 0.633 | 0.754 | 0.729 | 0.516 | 0.975 |

**Supplementary Table 6.** Different filter and radiomic features

| **Filter** | **Firstorder** | **GLCM** | **GLSZM** |
| --- | --- | --- | --- |
| Original  wavelet-LLH  wavelet-LHL  wavelet-LHH  wavelet-HLL  wavelet-HLH  wavelet-HHL  wavelet-HHH  wavelet-LLL | '10Percentile',  '90Percentile',  'Energy',  'Entropy',  'InterquartileRange',  'Kurtosis',  'Maximum',  'MeanAbsoluteDeviation',  'Mean',  'Median',  'Minimum',  'Range',  'RobustMeanAbsoluteDeviation',  'RootMeanSquared',  'Skewness',  'TotalEnergy',  'Uniformity',  'Variance' | \| 'Autocorrelation', \| \| --- \| \| 'ClusterProminence', \| \| 'ClusterShade', \| \| 'ClusterTendency', \| \| 'Contrast', \| \| 'Correlation', \| \| 'DifferenceAverage', \| \| 'DifferenceEntropy', \| \| 'DifferenceVariance', \| \| 'Id', \| \| 'Idm', \| \| 'Idmn', \| \| 'Idn', \| \| 'Imc1', \| \| 'Imc2', \| \| 'InverseVariance', \| \| 'JointAverage', \| \| 'JointEnergy', \| \| 'JointEntropy', \| \| 'MCC', \| \| 'MaximumProbability \| \| 'SumAverage', \| \| 'SumEntropy', \| \| 'SumSquares', \| | 'GrayLevelNonUniformityNormalized',  'GrayLevelVariance',  'HighGrayLevelZoneEmphasis',  'LargeAreaEmphasis',  'LargeAreaHighGrayLevelEmphasis',  'LargeAreaLowGrayLevelEmphasis',  'LowGrayLevelZoneEmphasis',  'SizeZoneNonUniformity',  'SizeZoneNonUniformityNormalized',  'SmallAreaEmphasis',  'SmallAreaHighGrayLevelEmphasis',  'SmallAreaLowGrayLevelEmphasis',  'ZoneEntropy',  'ZonePercentage',  'ZoneVariance' |
| **GLRLM** | **NGTDM** | **GLDM** | **SHAPE** |
| 'GrayLevelNonUniformity',  'GrayLevelNonUnifor'mityNormalized',  'GrayLevelVariance',  'HighGrayLevelRunEmphasis',  'LongRunEmphasis',  'LongRunHighGrayLevelEmphasis',  'LongRunLowGrayLevelEmphasis',  'LowGrayLevelRunEmphasis',  'RunEntropy',  'RunLengthNonUniformity',  'RunLengthNonUnifor'mityNormalized',  'RunPercentage',  'RunVariance',  'ShortRunEmphasis',  'ShortRunHighGrayLevelEmphasis',  'ShortRunLowGrayLevelEmphasis', | 'Busyness',  'Coarseness',  'Complexity',  'Contrast',  'Strength' | 'DependenceEntropy',  'DependenceNonUniformity',  'DependenceNonUniformityNormalized',  'DependenceVariance',  'GrayLevelNonUniformity',  'GrayLevelVariance',  'HighGrayLevelEmphasis',  'LargeDependenceEmphasis',  'LargeDependenceHighGrayLevelEmphasis',  'LargeDependenceLowGrayLevelEmphasis',  'LowGrayLevelEmphasis',  'SmallDependenceEmphasis',  'SmallDependenceHighGrayLevelEmphasis',  'SmallDependenceLowGrayLevelEmphasis', | 'Elongation',  'Flatness',  'LeastAxisLength',  'MajorAxisLength',  'Maximum2DDiameterColumn',  'Maximum2DDiameterRow',  'Maximum2DDiameterSlice',  'Maximum3DDiameter  'MeshVolume',  'MinorAxisLength',  'Sphericity',  'SurfaceArea',  'SurfaceVolumeRatio  VoxelVolume', |

**2. Supplementary Figures**


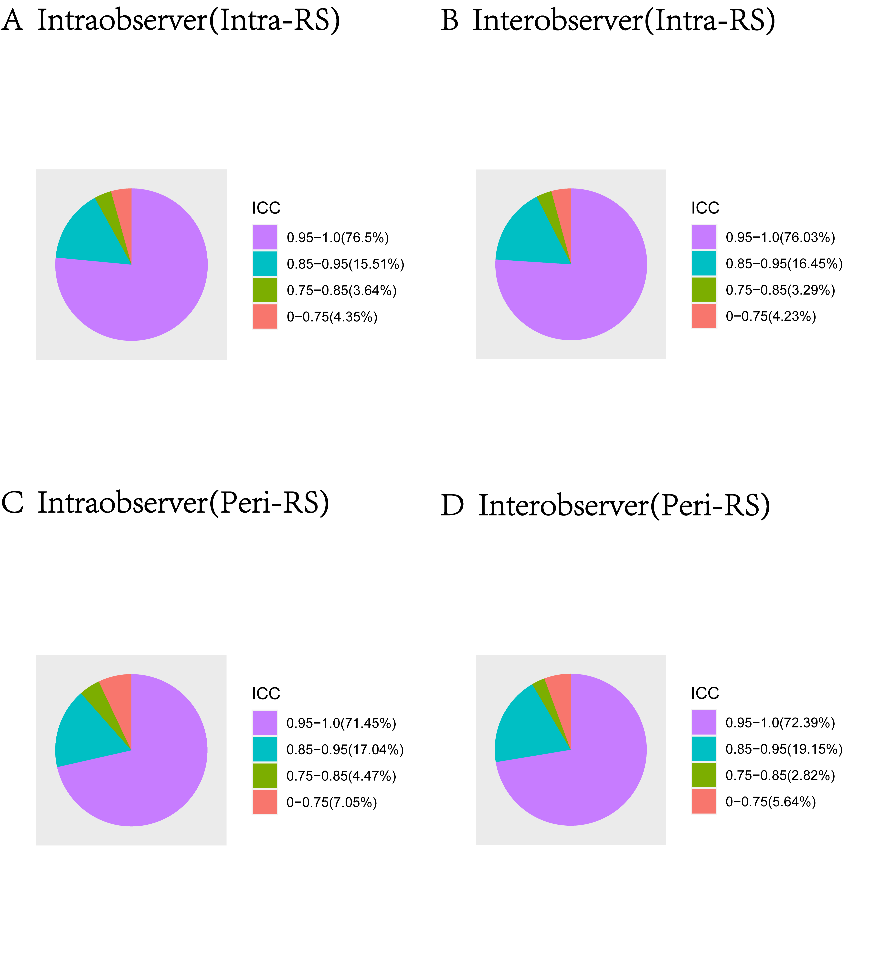


**Supplementary Figure 1.** Intra-observer (A, C) and inter-observer (B, D) ICC analysis. ICC：Intraclass correlation efficient；Intra-RS：intranodal features；Peri-RS：perinodal features


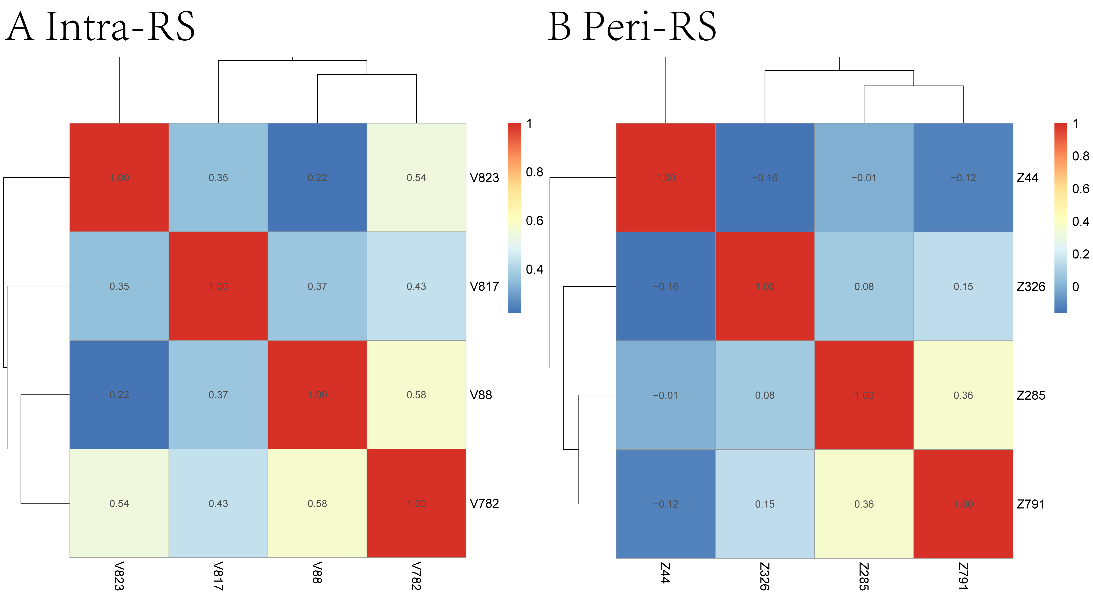


**Supplementary Figure 2.** Heat map of correlation coefficients for intranodal and perinodal features. V represents intranodular features, and Z represents perinodular features.


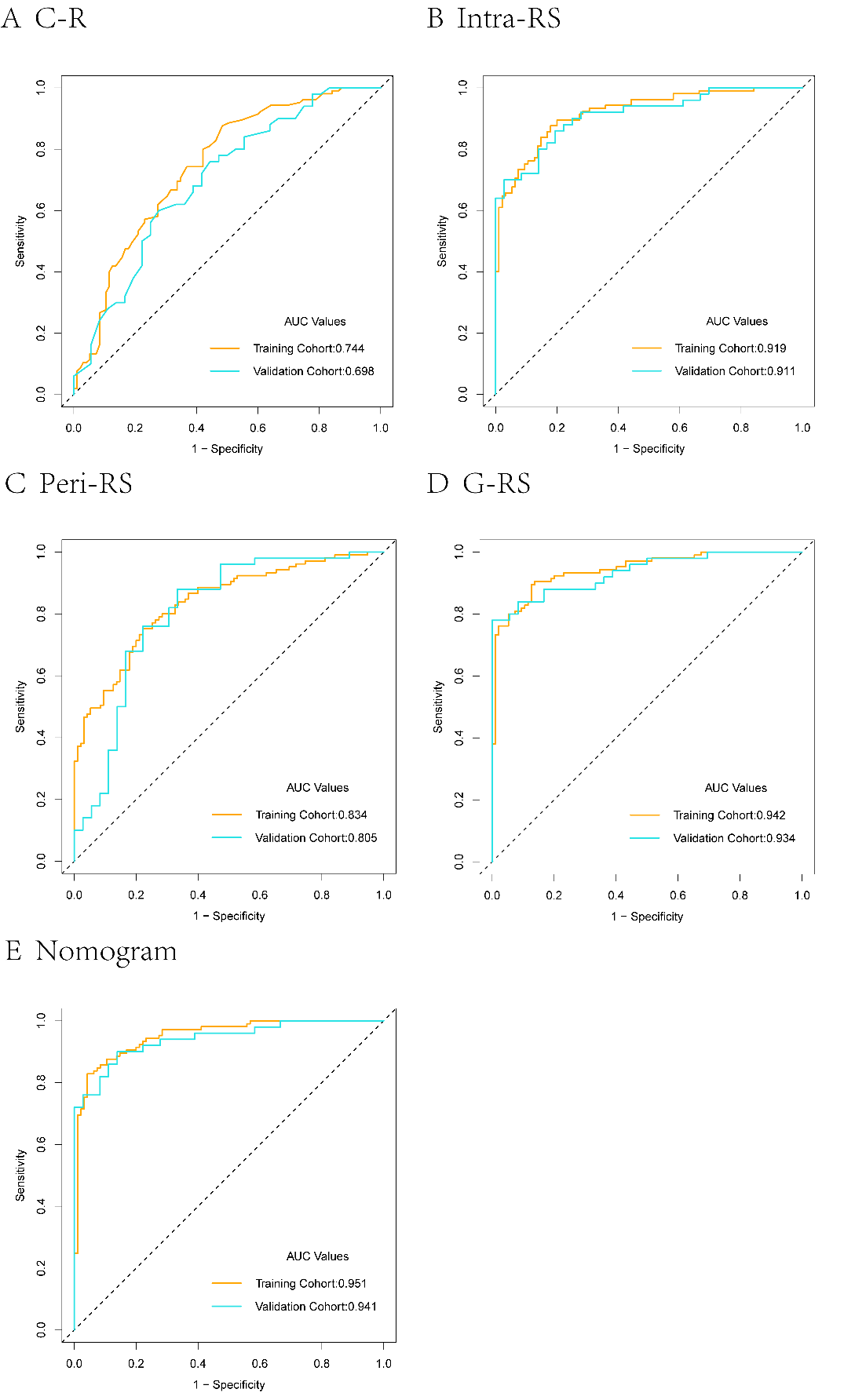


**Supplementary Figure 3.** ROC curves for 5 models. C-R: clinical radiology score; Intra-RS: intranodal radiomic score; Peri- RS: perinodal radiomic score; G- RS: intranodal plus perinodal radiomic score; Nomogram: Model for combined clinical radiology, intranodal, and perinodal radiomics


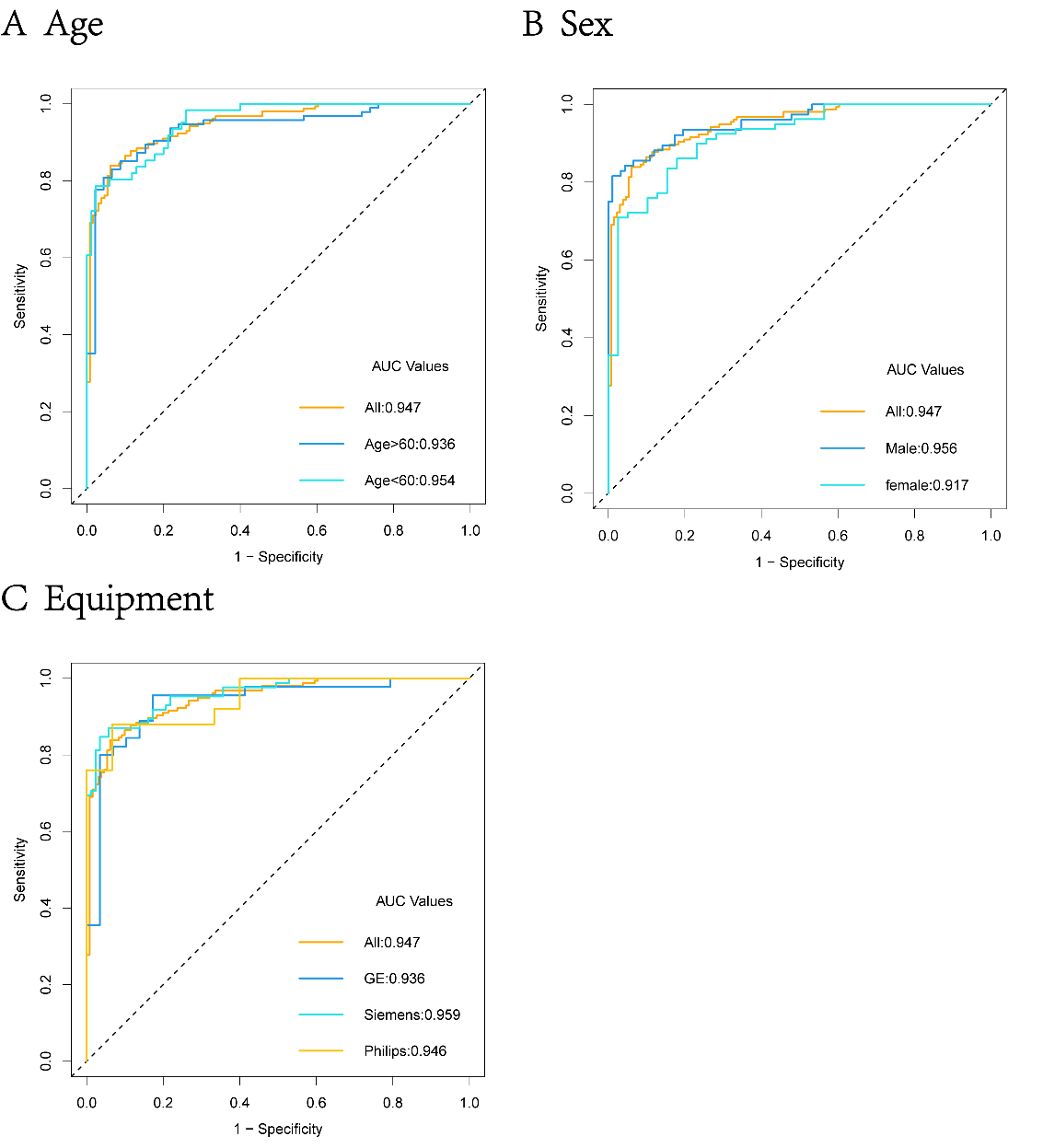


**Supplementary Figure 4.** ROC curves for subgroup analysis.
